# Dietary Fiber Intake Alters Gut Microbiota Composition but Does Not Improve Gut Wall Barrier Function in Women with Future Hypertensive Disorders of Pregnancy

**DOI:** 10.3390/nu12123862

**Published:** 2020-12-17

**Authors:** Kate I. Tomsett, Helen L. Barrett, Evelyn E. Dekker, Leonie K. Callaway, David H. McIntyre, Marloes Dekker Nitert

**Affiliations:** 1 School of Chemistry and Molecular Biosciences, The University of Queensland, St Lucia, QLD 4072, Australia; kate.tomsett@hotmail.com; 2 Mater Medical Research Institute, The University of Queensland, South Brisbane, QLD 4001, Australia; helen.barrett@mater.uq.edu.au (H.L.B.); h.d.mcintyre@uq.edu.au (D.H.M.); 3 Queensland Academy for Science, Mathematics and Technology, Toowong, QLD 4066, Australia; evelynedekker@gmail.com; 4 Women’s and Newborns, Royal Brisbane and Women’s Hospital, Herston, QLD 4029, Australia; Leonie.Callaway@health.qld.gov.au

**Keywords:** hypertension, dietary fiber, gut microbiota, pregnancy, zonulin, inflammation

## Abstract

Pregnancy alters the inflammatory state, metabolic hormones, and gut microbiota composition. It is unclear if the lower abundance of dietary fiber-fermenting, short-chain fatty acid-producing bacteria observed in hypertension also occurs in hypertensive disorders of pregnancy (HDP). This study investigated the relationship between dietary fiber intake and the gut microbiota profile at 28 weeks gestation in women who developed HDP in late pregnancy (*n* = 22) or remained normotensive (*n* = 152) from the Study of PRobiotics IN Gestational diabetes (SPRING). Dietary fiber intake was classified as above or below the median of 18.2 g/day. Gut microbiota composition was examined using 16S rRNA gene amplicon sequencing. The gut permeability marker zonulin was measured in a subset of 46 samples. In women with future HPD, higher dietary fiber intake was specifically associated with increased abundance of *Veillonella,* lower abundance of *Adlercreutzia, Anaerotruncus* and *Uncl. Mogibacteriaceae* and higher zonulin levels than normotensive women. Fiber intake and zonulin levels were negatively correlated in women with normotensive pregnancies but not in pregnancies with future HDP. In women with normotensive pregnancies, dietary fiber intake may improve gut barrier function. In contrast, in women who develop HDP, gut wall barrier function is impaired and not related to dietary fiber intake.

## 1. Introduction

Hypertensive disorders of pregnancy (HDP) complicate 5–10% of pregnancies [1]. The prevalence of HDP increases with maternal obesity [2]. Outside pregnancy, the gut microbiota is significantly less rich and diverse in patients with (pre)hypertension and shows increased abundance of the bacterial genera *Prevotella* and *Klebsiella* [3]. The composition of the gut microbiota is linked with metabolic health, inflammation, and pregnancy outcome [4,5,6]. The gut microbiota is profoundly altered during pregnancy along with hormonal, immunological, and metabolic changes [5], including increased body fat and decreased insulin sensitivity [7]. Decreased insulin sensitivity and increased adiposity are reportedly linked with inflammation at the intestinal mucosal epithelium, which can drive microbial dysbiosis [8]. The intestinal epithelium responds to a broad range of cytokines that impact on homeostasis and gut wall barrier integrity. Inflammatory cytokines compromise epithelial barrier function, and increase epithelial apoptosis and the release of antimicrobial peptides [6].

High dietary fiber intake promotes a rich and diverse gut microbiota outside pregnancy [9,10,11,12]. Fermentable fibers promote the growth of beneficial bacteria [13], which yield short-chain fatty acids (SCFAs), including acetate, propionate, and butyrate, as fermentation end products [10]. Higher fiber intake results in greater SCFA production [14]. These SCFAs lower colonic pH and thus prevent the growth of pathogenic bacteria and improve tight junction integrity of colonic epithelial cells [15]. This strengthens the integrity of the gut wall barrier and lowers gut permeability. Women who are overweight and obese in pregnancy have been reported to have decreased SCFA production and increased circulating lipopolysaccharide (LPS) levels, energy harvest, and insulin resistance [5,8]. Bacterial genera that have been reported to be associated with pregnancy and inflammation are *Collinsella, Anaerotruncus, Oscillospira*, and *Staphylococcus* [16,17]. Anti-inflammatory SCFA-producing bacterial genera reportedly associated with a healthy pregnancy include *Faecalibacterium, Bifidobacterium*, and *Veillonella* [18,19].

When evaluating increased gut permeability, it is important to consider that low-level inflammation increases throughout pregnancy [20]. As pregnancy progresses, the mucosal surface of the gut experiences low-grade inflammation with greater levels of pro-inflammatory cytokines, likely resulting in increased gut permeability. Maternal obesity and gestational diabetes mellitus (GDM) or an impaired gut wall barrier can shift the maternal inflammatory state from a physiological to an excessive level, resulting in further metabolic or vascular dysfunction. Vascular dysfunction of placental tissue can lead to serious complications, including fetal growth restriction and preeclampsia [21]. Metabolic dysfunction can lead to an array of complications, including increased cardiometabolic risks, hypertension, atherogenic dyslipidemia, and pre-term births [22]. It is unclear if dietary fiber intake affects the composition of the gut microbiota and/or gut barrier integrity in pregnancy and whether this is altered in women who develop hypertensive disorders of pregnancy (HDP) before the development of symptoms.

The aim of this study was to assess if fiber intake alters gut microbiota composition and gut permeability in a cohort of women with overweight and obesity in pregnancy, with and without future HDP, in late gestation (28 weeks) but prior to the development of HDP. To address this relationship, fecal microbiota profiles were assessed using 16S rRNA gene amplicon sequencing of women with variable fiber intake and correlated with levels of circulating gut permeability.

## 2. Materials and Methods

### 2.1. Study Population and Sample Collection

In this study, we included 22 normotensive (NT) and normoglycemic women with future HDP and 152 women without complications of pregnancy (COP). These were a subset of 174 women enrolled in the SPRING (Study of Probiotics in the Prevention of Gestational Diabetes Mellitus) study [23,24] (see Supplementary Materials Figure S1). The study was approved by the human research ethics committee of the Royal Brisbane and Women’s Hospital (HREC/11/QRBW/467) and The University of Queensland (201200080). All participants provided informed written consent. Inclusion in this subset was determined by completion of the study (from enrolment at 16 weeks to 28 weeks gestation) and provision of all data, including fecal samples and dietary information at both timepoints. Women with pre-existing glucose intolerance, inflammatory bowel disease or on medication known to affect glucose metabolism were excluded from participating in the SPRING study. Women diagnosed with preeclampsia, chronic hypertension, preeclampsia superimposed on chronic hypertension, and gestational hypertension diagnosed in routine clinical care were included in the future HDP group. One woman was prescribed labetalol for chronic hypertension. A sensitivity analysis indicated that removal of her data did not affect the results. All women developed HDP in late pregnancy after 34 weeks gestation. Dietary intake, stool samples, anthropometric measures, and blood samples were collected at 16 and 28 weeks gestation. Fecal samples were collected by the participant at home following an instruction sheet and refrigerated until storage at −80 °C within 24 h. Fiber intake was recorded using the Victorian Cancer Council’s food frequency questionnaire (FFQ) [25] asking women to record their dietary intake during pregnancy. This questionnaire is comprised of a food list with 74 items and 10 frequency responses ranging from ‘never’ to ‘3 or more times per day’. To determine portion size, women were shown three images of food served on a plate and asked to identify their intake. Food composition data used to calculate nutrients are from NUTTAB95. DQESv2 analysis was performed providing information including daily kilojoule intake, and detailed macronutrient and micronutrient profiles. Dietary fiber intake was classified as below (lower) or above (higher) the median dietary fiber intake of 18.2 g/day (interquartile range 13.89–23.2 g/day) in this cohort.

### 2.2. Fecal DNA Extraction

DNA was isolated from stool samples using 0.25 g of stool thawed overnight at 4 °C. The repeated bead beating and column method was used [26], employing the TissueLyser II (Qiagen) with sterile zirconia beads (a mixture of 0.1 and 0.5 mm diameter). The stool samples were lysed by bead beating for 3 min at 30 Hz. To extract and purify bacterial DNA, the Qiagen AllPrep DNA extraction kit was used. DNA concentration for each sample was determined using a Nanodrop ND 1000 spectrophotometer.

### 2.3. Bioinformatics Analysis

DNA sequences were retrieved using 16S rRNA gene amplicon sequencing by the Illumina MiSeq system at the University of Queensland Australian Centre for Ecogenomics. The V6–V8 region of the 16S rRNA gene were amplified from purified genomic DNA by using the primers 926F (5′-TCG TCG GCA GCG TCA GAT GTG TAT AAG AGA CAG AAA CTY AAA KGA ATT GRC GG-3′) and (5′-GTC TCG TGG GCT CGG AGA TGT GTA TAA GAG ACA GAC GGG CGG TGW GTR C-3′) 1392R with overhang adapters. Each product was 466 base pairs long. V6–V8 amplicons were cleaned using AMPure XP beads. This removed unbound primers, primer dimer species, and nucleotides.

Taxonomy was identified using QIIME2.0 [27]. Primer sequences were removed using ‘cutadapt’. All data sequences were imported into QIIME2.0 with a manifest to assign each sequence a sample name. Sequences were adjusted, denoised, and exported as a feature table. A taxonomy file was created with QIIME2.0 using the GreenGenes database (version 13_8) [28] with an identity threshold of 99%.

### 2.4. Zonulin Assay

Circulating serum zonulin levels were assayed in duplicate at 16 and 28 weeks gestation by ELISA (Abcam human haptoglobin kit cat no 219048, Melbourne, Australia) according to the manufacturer’s recommendations. Samples from 46 women, at 16 and 28 weeks gestation, were thawed overnight at 4 °C. The samples from 16 weeks were diluted to 1:8000 for the future HDP samples and 1:1000 for the healthy control samples whereas at 28 weeks gestation, all samples were diluted 1:8000 in assay diluent. The clinical characteristics of these women are presented in Supplementary Materials Table S1.

### 2.5. Data Analysis

Lower and higher fiber conditions were separated according to the group median fiber intake of 18.2 g/day or as quartile 1 < 13.9 g/day vs. quartile 4 > 23.1 g/day. Comparisons between the two fiber groups were performed using the Mann–Whitney U test. Analysis was performed using GraphPad Prism 8 software and a *p*-value less than 0.05 was considered statistically significant. Analysis of the gut microbiota composition was performed with the Calypso software suite [29] at the genus level. Gut microbiota alpha diversity was assessed using Chao1 and the Shannon indices. These provide an indication diversity and evenness (Shannon) or diversity only (Chao1) of the bacterial genera. Beta diversity was assessed using Anosim, unsupervised hierarchical clustering analysis (PCoA), supervised hierarchical clustering by redundancy analysis (RDA), and Adonis tests. These provided a measure of microbial diversity between patients. Comparisons of the abundances of specific bacterial genera were performed with Wilcoxon rank tests. Associations between metadata (fiber intake, biochemical measurements and clinical data) and taxa abundance were evaluated using the Spearman rank correlation coefficient. Data were not corrected for multiple testing due to the relatively small sample size of the future HDP cohort after subdividing into higher and lower fiber intake.

## 3. Results

### 3.1. Overall Gut Microbiota Diversity

**Figure 1:**
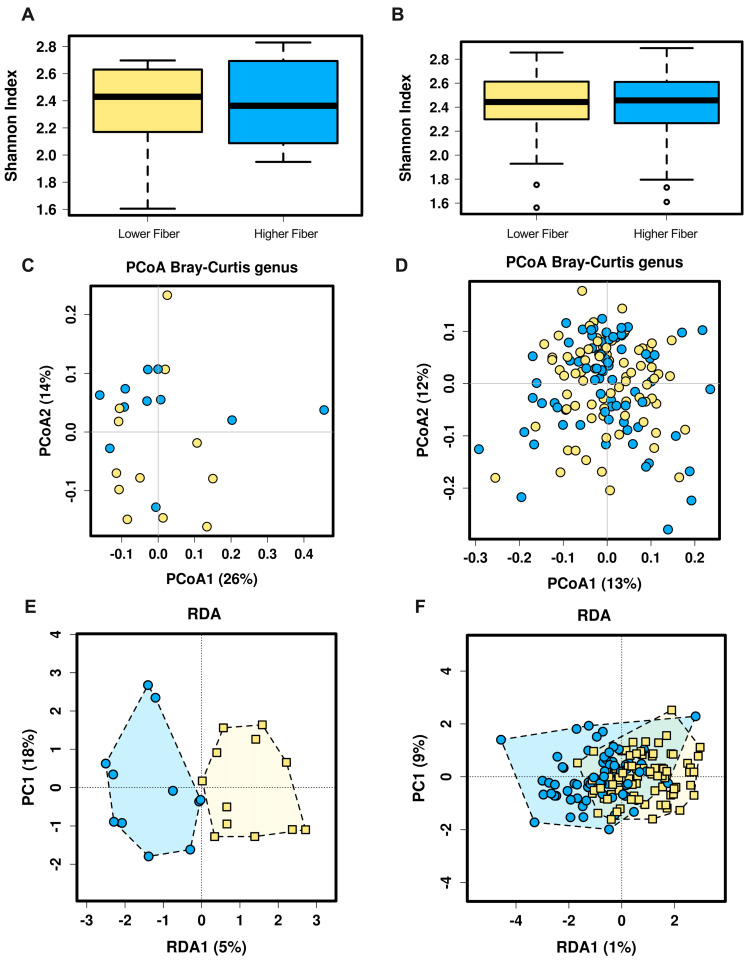
Alpha and beta diversity of the gut microbiota in women with HDP and NT. Alpha diversity as measured by the Shannon index in women with future HDP (**A**) and NT women (**B**). Beta diversity as measured by unsupervised hierarchical clustering by PCoA in women with HDP (**C**) and NT women (**D**). Beta diversity as measured by supervised hierarchical clustering by RDA in women with HDP (**E**) and NT women (**F**). Yellow symbols, lower fiber; blue symbols, higher fiber. HDP = Hypertensive disorders of pregnancy. NT = Normotensive.

Clinical characteristics of participants at 28 weeks gestation are presented in Table 1 and Table 2. Twenty-two women had future HDP (*n* = 10, lower fiber; *n* = 12, higher fiber), with the majority having normal blood pressure at the time of fecal sampling. Of the 22 women in the future HDP group, 16 were diagnosed with preeclampsia and 5 with gestational hypertension in late pregnancy [30]. In total, 152 women had a normotensive pregnancy (*n* = 79, lower fiber; *n* = 73, higher fiber). There was no difference in higher and lower fiber intake at 28 weeks gestation between women with (*p* = 0.52) and without (*p* = 1.0) future HDP, nor in alpha diversity of the gut microbiota (results not shown). When comparing bacterial richness in women with higher and lower fiber intake in future HDP and normotensive conditions, the Chao1 indices were similar, indicating that rare bacterial genera were not more or less abundant with varying daily fiber intake (*p* = 0.44 and *p* = 0.91, respectively). Additionally, accounting for both abundance and evenness of the genera present with the Shannon index also showed no effect of fiber intake in women with future HDP or normotensive pregnancies (Figure 1A,B, respectively).

Beta diversity, e.g., the diversity of the gut microbiota between groups, was measured using PCoA and RDA analysis in women with and without future HDP. Unsupervised hierarchical clustering analysis by PCoA showed that little of the variation in beta diversity was due to fiber intake as indicated by the lack of separation of the individuals into distinct groups (Figure 1C,D). Supervised clustering analysis of beta diversity in women with future HDP by RDA demonstrated altered beta diversity in women with lower fiber intake, although not significantly (*p* = 0.11, Figure 1E). This trend was not observed in normotensive women (Figure 1F).

### 3.2. Gut Microbiota Composition

**Figure 2:**
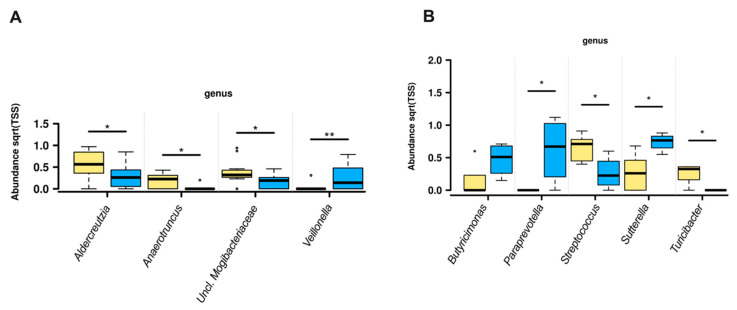
Comparison of genus abundance in women with future HDP at 28 weeks gestation with low and high fiber intake. (**A**) Genus abundance with fiber intake below (lower fiber; yellow boxes) or above (higher fiber; blue boxes) the median (18.2 g/day). (**B**) Genus abundance with fiber intake in the lowest (Q1; <13.9 g/day; yellow boxes) and highest (Q4; >23.1 g/day; blue boxes) of fiber intake. * = *p* < 0.05, ** = *p* < 0.01.

Investigation of the specific genera that were altered by dietary fiber intake showed that in women with future HDP and higher fiber intake, *Veillonella* abundance was increased whereas *Adlercreutzia, Anaerotruncus,* and unclassified *Mogibacteriaceae* abundances were decreased (Figure 2A). Different genera abundance was observed comparing the highest and lowest quartiles of fiber intake. When >23.1 g/day (*n* = 6) of fiber was consumed, *Butyricimonas, Paraprevotella*, and *Sutterella,* abundance was increased whereas if fiber consumption was <13.9 g/day (*n* = 8), *Streptococcus* and *Turicibacter* abundance was increased (Figure 2B).

**Figure 3:**
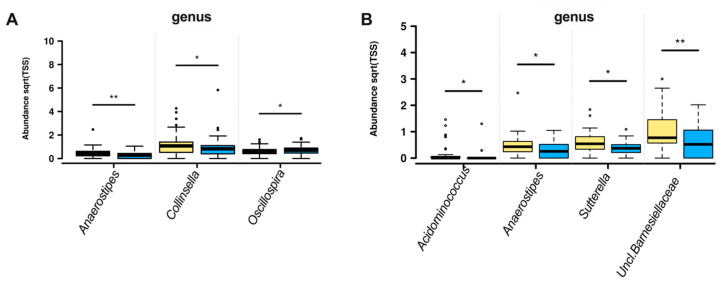
Comparison of genera abundance at 28 weeks gestation in women with lower and higher fiber intake who remain normotensive. (**A**) Genus abundance with fiber intake below (lower fiber; yellow boxes) or above (higher fiber; blue boxes) the median (18.2 g/day). (**B**) Genus abundance with fiber intake in the lowest (Q1; <13.9 g/day; yellow boxes) and highest (Q4; >23.1 g/day; blue boxes) of fiber intake. * = *p* < 0.05, ** = *p* < 0.01.

In women who remained normotensive throughout pregnancy, higher fiber intake was associated with increased *Oscillospira* abundance and lower abundance of *Anaerostipes* and *Collinsella* (Figure 3A). Further separation of fiber intake using the highest and lowest quartiles (>23.1 and <13.9 g/day, respectively) demonstrated that high fiber intake was associated with lower abundance of *Acidaminococcus, Anaerostipes, Sutterella*, and unclassified *Barnesiellaceae* (Figure 3B).

In women with future HDP, fiber intake was positively correlated with *Faecalibacterium* abundance (rho = 0.40, *p* = 0.03) and negatively correlated with *Collinsella* (r = −0.41, *p* = 0.02)*, Oscillospira* (r = −0.39, *p* = 0.03) and *Anaerotruncus* (r = −0.45, *p* = 0.01) abundance. In women without future HDP, *Anaerostipes* (r = −0.22, *p* = 0.01) and unclassified *Barnesiellaceae* (r = −0.20, *p* = 0.01) abundance was negatively correlated with fiber intake whereas *Collinsella* (r = −0.14, *p* = 0.09) abundance only trended towards a negative correlation with fiber intake.

### 3.3. Gut Permeability

**Figure 4:**
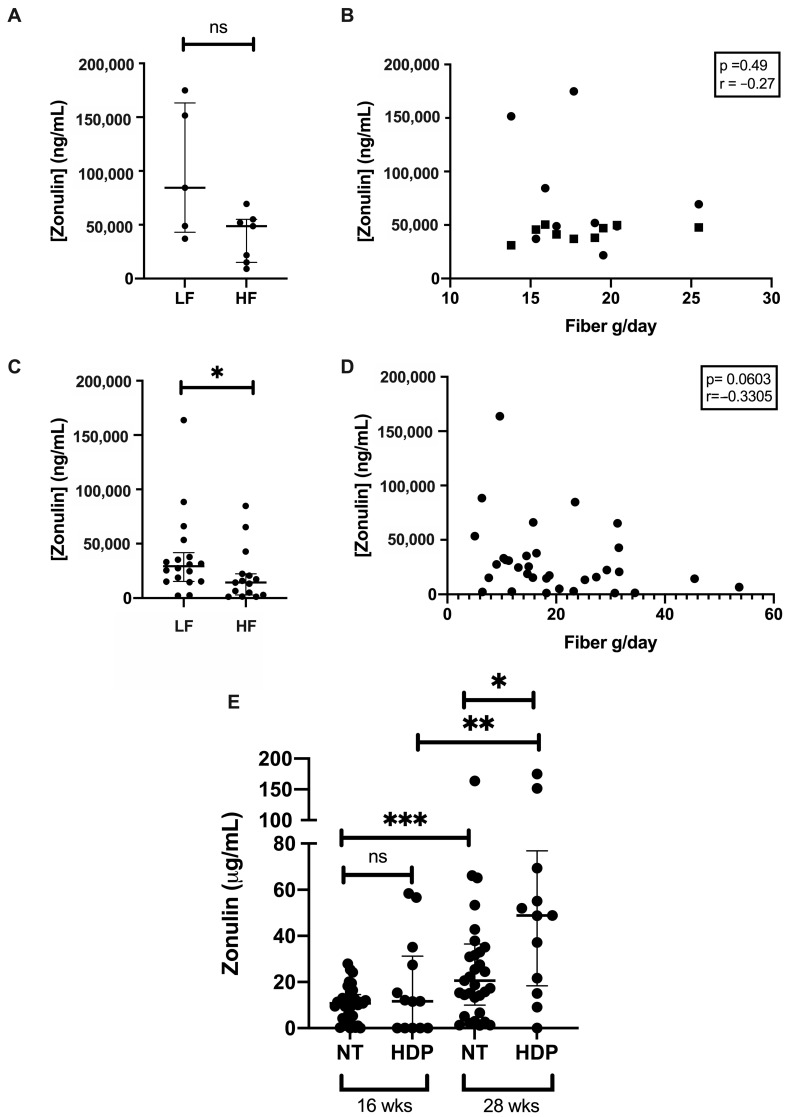
Serum zonulin concentrations in HDP and NT pregnancies baseline and 28 weeks gestation. Serum zonulin concentrations in lower (LF) and higher fiber (HF) groups in future HDP (**A**) and NT women (**C**). (**B**) Correlation of dietary fiber intake and zonulin concentrations in HDP (**B**) and NT women (**D**). (**E**) Serum zonulin concentration in HDP and NT pregnancies at baseline and 28 weeks gestation. HDP = Hypertensive disorders of pregnancy. NT = Normotensive. * = *p* < 0.05, ** = *p* < 0.01, *** = *p* < 0.001.

Women with future HDP had similar levels of circulating zonulin in the lower and higher fiber groups (*p* = 0.11; Figure 4A) and showed no correlation between fiber intake and zonulin levels (Figure 4B). In contrast, in women who remained normotensive, serum zonulin concentrations were significantly lower in women with higher fiber intake compared with lower fiber intake at 28 weeks gestation (*p* = 0.036) (Figure 4C) and there was a trend toward a negative correlation between fiber intake and zonulin levels (Figure 4D). When comparing zonulin levels in women with and without future HDP over gestation, there were no differences at 16 weeks gestation (Figure 4E, *p* = 0.75). At 28 weeks gestation, zonulin levels increased in both groups and the future HDP group now had significantly higher zonulin levels than the control group (Figure 4E, *p* = 0.012). In women with future HDP but not in women who remained normotensive in pregnancy, zonulin concentrations significantly increased as pregnancy progressed (Figure 4E, *p* = 0.004).

## 4. Discussion

This study showed that dietary fiber intake is associated with changes in genus abundance but not the overall diversity of the gut microbiota in women with and without future HDP. In women with normotensive pregnancies, higher dietary fiber intake is associated with decreased gut permeability; however, this beneficial association was not present in women with future HDP and who showed higher levels of gut permeability compared to women who had a normotensive pregnancy.

### 4.1. Genera Diversity and Abundance with Fiber Intake

Gut microbiota diversity is hypothesized to be already reduced in pregnant women who are overweight or obese [16] and our data suggests that fiber intake or lack thereof did not affect this further. Fiber intake was associated with reduced *Collinsella* abundance in the women who remained normotensive in pregnancy. We have previously reported that greater *Collinsella* abundance is positively correlated with insulin resistance and greater gut inflammation [17]. Insulin resistance increases over normal gestation and may be partially determined by the abundance of *Collinsella.* If increased fiber intake or the decreased dietary fat intake that often is present in a high-fiber diet reduces *Collinsella* abundance, this may help to reduce insulin resistance. *Oscillospira* abundance increased in women who remained normotensive and who had higher fiber intake although it was not significantly different when comparing the extremes of fiber intake. *Oscillospira* abundance has been shown to decrease with high fiber intake as its preferred substrates (oligosaccharides, glycerol-phosphate, and polyols) [31] are likely lower in a high-fiber diet [32,33]. *Oscillospira* abundance is key to butyrate production and supports the growth of the butyrate producers *Faecalibacterium* and *Coprococcus* [31]. It is unclear why *Oscillospira* abundance increased in the higher fiber intake group; it is possible that other determinants, such as BMI, intestinal transit time, and immune status, play a larger role than fiber intake in pregnancy [31].

Higher-fiber diet increased the abundance of bacteria with known anti-inflammatory functions in women with future HDP, including *Butyricimonas* and *Veillonella*. *Butyricimonas* is a butyrate producer that enhances the intestinal barrier integrity by stimulating the assembly of tight junction proteins [19]. This potentially reduces the transport of gut lumen content across the barrier, thus contributing to reduced systemic inflammation [18,34]. *Veillonella* is a lactate producer and may lead to lower circulating cholesterol levels and control of intestinal infections through its bactericidal effect on pathogenic bacteria [35]. Although *Veillonella* is not ubiquitously present, its abundance has been negatively correlated with concentrations of the blood glucose regulators C-peptide, insulin, and visfatin [11,36]. The increased *Veillonella* abundance in women with higher dietary fiber intake may contribute to improved insulin sensitivity.

### 4.2. Gut Wall Barrier Function Across Gestation

Zonulin levels increased over gestation in both groups although to a larger extent in women with future HDP, who had significantly higher zonulin levels at 28 weeks gestation. This could indicate that the tight junctions of gut epithelia become weaker across gestation, possibly contributing to the increased systemic inflammation [37]. It also suggests that gut wall barrier function is compromised before blood pressure starts to rise in women with future HDP. Whether strategies other than dietary fiber intake to improve gut wall barrier function can improve gut wall barrier function in women with future HDP needs to be investigated.

### 4.3. Gut Wall Barrier Function and Fiber Intake

Women who remain normotensive throughout pregnancy have significantly lower serum zonulin with a higher-fiber diet. Higher zonulin concentrations are associated with dysregulation of the mucosal barrier leading to increased passage of antigens and other macromolecules from the external environment into the host, initiating inflammation and immune activation [38]. It appears that, similar to what has been reported outside pregnancy [39], the relationship between higher fiber intake, greater abundance of SCFA-producing bacteria, and strong gut barrier integrity is present in normotensive pregnancies. However, this was not the case in women without future HDP, who did not show a relationship between fiber intake and zonulin levels. This may be because other factors determining gut wall barrier status, including immune stimulation, have a stronger effect than dietary fiber intake.

### 4.4. Limitations and Future Directions

The major limitation of this study is the relatively small size of the future HDP cohort. In addition, women who eat more fiber also have a higher caloric intake, consuming more macro- and micronutrients in general. However, fiber still appears to be beneficial, indicating that it may be the absolute amount of fiber and not the proportion of fiber as part of the whole diet that appears beneficial. Less than 18% of women in this study achieved a daily fiber intake above the minimum recommended daily requirement of 25 g/day [40]. This is representative of the fiber intake of the general obstetric population; however, a true representation of a high-fiber diet was lacking. Women with future HDP did not have lower fiber intake compared to women who remained normotensive. Their gut microbiota is not poorly established, but the same fiber intake appears to be associated with fewer health benefits compared to normotensive women. Metagenomic analysis of the gut microbiota would shed light on the functional capacity of the microbiota, which may add mechanistic insights into how the bacteria contribute to the increased gut permeability of women with future HDP.

## 5. Conclusions

In conclusion, fiber intake is associated with altered composition of the gut microbiota. Higher fiber intake increases the abundance of bacteria associated with decreased inflammation. Higher fiber intake is associated with a lower increase in gut permeability as pregnancy progresses in women who remain normotensive but does not affect gut permeability in women with future HDP. This suggests that fiber interventions may not prevent the development of HDP through improving gut barrier function.

## Figures and Tables

**Table 1:** Participant Characteristics Lower and Higher Fiber Groups at 28 weeks gestation.

Fiber Condition	Hypertensive Disorders of Pregnancy			Normotensive		
	Lower Fiber	Higher Fiber	*p*-Value	Lower Fiber	Higher Fiber	*p*-Value
N	10	12	NA	79	73	NA
Maternal age (years)	33 (1.34)	38 (1.43)	**0.05**	32 (0.55)	33 (0.53)	0.67
Maternal BMI (kg/m^2^)	34.9 (2.36)	34.2 (1.28)	0.77	34.9 (0.62)	33.9 (0.67)	0.31
Excess weight gain (n) (Y/N/No Data)	2/8/0	6/6/0	0.20	24/44/11	31/31/11	0.12
Ethnicity			NA			0.06
Caucasian *n* (%)	90.0	91.7		86.1	90.4	
Indian *n* (%)	0	0		1.3	0	
Asian *n* (%)	0	8.3		7.6	0	
Other *n* (%)	10.0	0		5.1	9.6	
SBP^ (mmHg)	114 (2.10)	115 (4.39)	0.87	111 (1.02)	109 (0.98)	0.08
DBP^ (mmHg)	70 (2.34)	70 (2.93)	0.90	66 (0.86)	65 (0.87)	0.50
Fasting glucose^ (mmol/L)	4.3 (0.12)	4.5 (0.07)	0.24	4.3 (0.05)	4.3 (0.05)	0.81
C-Peptide^ (nmol/L)	1.1 (0.16)	0.9 (0.08)	0.39	0.9 (0.04)	0.8 (0.04)	**0.03**
Insulin^ (mU/L)	11.2 (1.22)	10.0 (1.78)	0.60	10.8 (0.68)	8.7 (0.54)	**0.02**
Total cholesterol^ (mmol/L)	6.9 (0.27)	6.2 (0.43)	0.21	6.6 (0.14)	6.7 (0.12)	0.34
Circulating Triglycerides^ (mmol/L)	2.6 (0.25)	1.9 (0.23)	0.08	2.1 (0.10)	2.0 (0.08)	0.48
Daily Energy Intake^ (kJ)	5351 (430)	6880 (278)	**0.0088**	5307 (128)	8022 (221)	**<0.0001**
Fiber intake^ (g/day)	12.8 (0.87)	22.8 (1.18)	**<0.0001**	13.6 (0.3)	25.4 (0.8)	**<0.0001**
Energy-corrected fiber intake^ (mg/kJ)	2.0 (0.11)	2.7 (0.20)	**0.005**	2.6 (0.1)	3.0 (0.1)	**<0.0001**
Carbohydrates (g/day)	122.7 (11.76)	171.1 (6.31)	**0.0003**	131.9 (3.4)	215.1 (6.7)	**<0.0001**
Starch (g/day)	62.8 (7.54)	86.3 (4.58)	**0.018**	66.6 (2.1)	116.6 (4.6)	**<0.0001**
Protein (g/day)	71.1 (6.74)	80.0 (5.14)	0.31	62.4 (2.0)	99.4 (3.4)	**<0.0001**
Total Fats (g/day)	56.6 (5.72)	71.6 (4.59)	**0.05**	55.2 (1.6)	88.1 (3.2)	**<0.0001**
Saturated Fats (g/day)	24.2 (2.47)	30.8 (2.75)	0.08	24.6 (0.8)	38.4 (1.5)	**<0.0001**
Fetal Sex (F/M)	4/5/1	7/5/0	0.71	37/26	28/31	0.28
Birth weight (g)	3528 (91.37)	3511 (129.7)	0.91	3605 (82)	3572 (64)	0.75

Results presented as mean with the standard error of the mean (SEM) unless otherwise mentioned. Comparisons between groups were performed with unpaired Student t-tests. ^Data was collected at 28 weeks gestation; BMI = Body Mass Index, SBP = Systolic Blood Pressure, DBP = Diastolic Blood Pressure. Bolded numbers represent significant differences.

**Table 2:** Participant Characteristic HDP vs. Normotensive and HDP with no GDM vs. Normotensive Groups at 28 weeks gestation.

	HDP No GDM	Normotensive	*p*-Value
N	22	152	NA
Maternal age (years)	36 (1.1)	35 (0.4)	0.62
Maternal BMI (kg/m^2^)	34.4 (1.3)	34.4 (0.5)	0.99
Ethnicity			0.94
Caucasian *n* (%)	20 (91)	134 (88)	
Indian *n* (%)	0 (0)	1 (<1)	
Asian *n* (%)	1 (4.5)	6 (4)	
Other *n* (%)	1 (4.5)	11 (7)	
SBP (mmHg)	114 (2.5)	110 (0.7)	**0.04**
DBP (mmHg)	70 (1.9)	66 (0.6)	**0.01**
Fasting glucose (mmol/L)	4.4 (0.1)	4.3 (0.0)	0.24
C-Peptide (nmol/L)	1.0 (0.1)	0.9 (0.0)	0.13
Insulin (mU/L)	11.2 (1.2)	9.8 (0.4)	0.26
Total cholesterol (mmol/L)	6.5 (0.3)	6.6 (0.1)	0.65
Circulating Triglycerides (mmol/L)	2.2 (0.2)	2.1 (0.1)	0.49
Daily Energy Intake (kJ)	6185 (293)	6512 (166)	0.46
Fiber intake (g/day)	18.3 (1.3)	19.3 (0.6)	0.58
Energy-corrected fiber intake (mg/kJ)	3.0 (0.2)	2.8 (0.1)	0.29
Carbohydrates (g/day)	149.1 (8.1)	171.9 (5.0)	0.09
Starch (g/day)	72.3 (4.9)	90.59 (3.2)	**0.03**
Protein (g/day)	75.9 (4.2)	80.2 (2.5)	0.52
Total Fats (g/day)	64.9 (3.9)	71.67 (2.2)	0.27
Saturated Fats (g/day)	27.8 (2.0)	31.2 (1.0)	0.21
Fetal Sex (F/M)	11/10	65/57	>0.99
Birth weight (g)	3518 (82)	3589 (52)	0.58

Results presented as mean with the standard error of the mean (SEM) unless otherwise mentioned. Comparisons between groups were performed with unpaired Student t-tests. BMI = Body Mass Index; SBP = systolic blood pressure; DBP = diastolic blood pressure. Bolded numbers represent significant differences.

## Supplementary Materials

The following are available online at https://www.mdpi.com/2072-6643/12/12/3862/s1, Figure S1: Study overview, Table S1: Participant characteristics HDP and NT subgroup for zonulin analysis.
