# Supplementary material for: Dietary Fiber Intake Alters Gut Microbiota Composition but Does Not Improve Gut Wall Barrier Function in Women with Future Hypertensive Disorders of Pregnancy
Source: Nutrients. 2020 Dec 17;12(12):3862. doi: 10.3390/nu12123862 (PMC7766248; doi:10.3390/nu12123862)
Supplement: Supplementary file 1 [file nutrients-12-03862-s001.pdf]

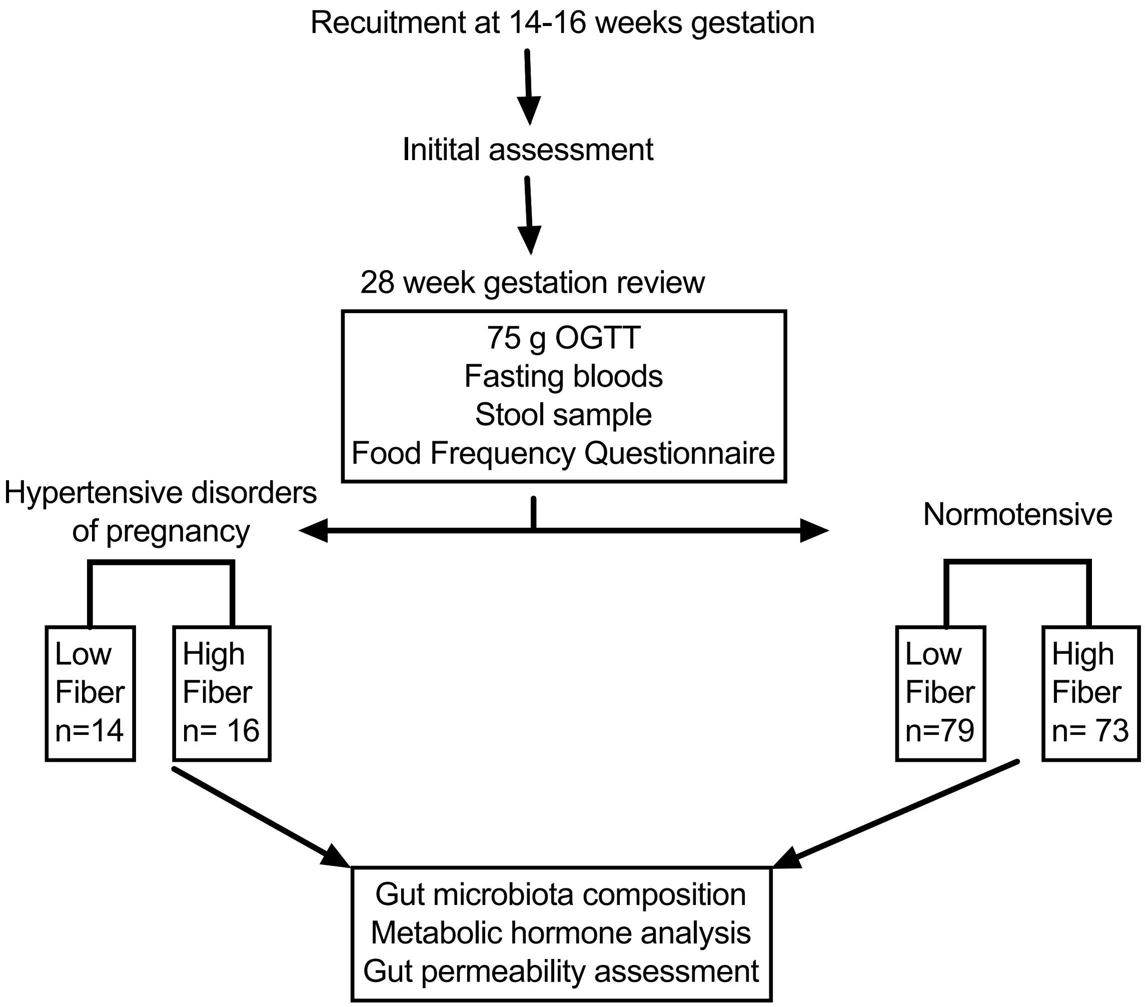

Figure 1. Study overview.

Table 1. Participant Characteristic HDP vs Normotensive Groups in the subgroup analysed for metabolic hormones and zonulin levels at 28 weeks.

	**Hypertensive Disorders of Pregnancy**	**Normotensive**	**p-value**
N	19	36	NA
Maternal age (years)	36.7 (1.4)	34.4 (029)	0.16
Maternal baseline BMI (kg/m^2)	35.9 (1.6)	32.7 (1.1)	0.09
SBP (mmHg)	118 (2.6)	110 (1.5)	**0.006**
DBP (mmHg)	73 (1.9)	65 (1.3)	**0.002**
Fasting glucose (mmol/L)	4.5 (0.1)	4.2 (0.1)	**0.005**
GDM N (%)	6 (32)	0 (0)	**0.001**
Total cholesterol (mmol/L)	6.3 (0.3)	6.5 (0.2)	0.69
Circulating Triglycerides (mmol/L)	2.3 (0.2)	2.1 (0.2)	0.48
Daily Energy Intake (kJ)	6001 (300)	7168 (444)	0.09
Fiber intake (g/day)	18.2 (1.2)	21.0 (1.8)	0.30
Energy-corrected fiber intake (mg/kJ)	2.9 (0.2)	2.9 (0.2)	0.60
Low Fiber/High Fiber (N)	10/8*	19/17	1.0
Carbohydrates (g/day)	143.9 (7.5)	180.7 (11.4)	**0.04**
Starch (g/day)	72.3 (4.5)	97.9 (6.8)	**0.01**
Protein (g/day)	69.9 (4.6)	82.3 (5.6)	0.16
Total Fats(g/day)	64.9 (3.9)	74.2 (5.0)	0.23
Saturated Fats (g/day)	28.3 (1.8)	32.1 (2.4)	0.30

Results presented as mean with the standard error of the mean (SEM) unless otherwise mentioned. Comparisons between groups were performed with unpaired Student t-tests, apart from rate of GDM which was assessed with Fisher's exact test. *, data missing from one individual; BMI, Body Mass Index; SBP, systolic blood pressure; DBP, diastolic blood pressure.
